# A History of Ashes: An 80 Year Comparative Portrait of Smoking Initiation in American Indians and Non-Hispanic Whites—the Strong Heart Study

**DOI:** 10.3390/ijerph10051747

**Published:** 2013-05-02

**Authors:** Raymond Orr, Darren Calhoun, Carolyn Noonan, Ron Whitener, Jeff Henderson, Jack Goldberg, Patrica Nez Henderson

**Affiliations:** 1 School of Social and Political Sciences, University of Melbourne, Level 4, John Medley Building, Parkville 3010, Victoria, Australia; 2 MedStar Research Institute, Phoenix, AZ 85016, USA; E-Mail: darren.calhoun@medstar.net; 3 Partnerships for Native Health, University of Washington, Seattle, WA 98101, USA; E-Mails: cnoonan@u.washington.edu (C.N.); ronw@u.washington.edu (R.W.); jack.goldberg@va.gov (J.G.); 4 Black Hills Center for American Indian Health, Rapid City, SD 57701, USA; E-Mails: jhenderson@bhcaih.org (J.H.); pnhenderson@bhcaih.org (P.N.H.)

**Keywords:** Indians, North American, smoking, age of Initiation, cigarette use, health disparities

## Abstract

The consequences of starting smoking by age 18 are significant. Early smoking initiation is associated with higher tobacco dependence, increased difficulty in smoking cessation and more negative health outcomes. The purpose of this study is to examine how closely smoking initiation in a well-defined population of American Indians (AI) resembles a group of Non-Hispanic white (NHW) populations born over an 80 year period. We obtained data on age of smoking initiation among 7,073 AIs who were members of 13 tribes in Arizona, Oklahoma and North and South Dakota from the 1988 Strong Heart Study (SHS) and the 2001 Strong Heart Family Study (SHFS) and 19,747 NHW participants in the 2003 National Health Interview Survey. The participants were born as early as 1904 and as late as 1985. We classified participants according to birth cohort by decade, sex, and for AIs, according to location. We estimated the cumulative incidence of smoking initiation by age 18 in each sex and birth cohort group in both AIs and NHWs and used Cox regression to estimate hazard ratios for the association of birth cohort, sex and region with the age at smoking initiation. We found that the cumulative incidence of smoking initiation by age 18 was higher in males than females in all SHS regions and in NHWs (*p* < 0.001). Our results show regional variation of age of initiation significant in the SHS (*p* < 0.001). Our data showed that not all AIs (in this sample) showed similar trends toward increased earlier smoking. For instance, Oklahoma SHS male participants born in the 1980s initiated smoking before age 18 less often than those born before 1920 by a ratio of 0.7. The results showed significant variation in age of initiation across sex, birth cohort, and location. Our preliminary analyses suggest that AI smoking trends are not uniform across region or gender but are likely shaped by local context. If tobacco prevention and control programs depend in part on addressing the origin of AI smoking it may be helpful to increase the awareness in regional differences.

## 1. Introduction

The prevalence of cigarette smoking among American Indians (AI) is among the highest of all ethnic groups in the U.S. [1,2], and if current trends continue, it is estimated that two in every five AIs will die from tobacco related illness [3]. According to the U.S. Surgeon General, 88% of all first time cigarette use occurs before age 18 and 99% by 26 [4]. The national estimate for AI smoking is 42% in males and 38% in females, which is double that of non-Hispanic whites (NHWs) [5]. Although the prevalence of AI smoking is high, smoking rates vary dramatically by tribe and reservation, with estimates as high as 73% among the Northern Plains [6]. Substantial evidence has established that smoking rates have declined among the general population since the early 1980s, though the decrease appears to be less dramatic among AIs [4,7]. 

A host of reasons may explain the high smoking rates among AIs, including traditional use of tobacco, socioeconomic disparities, discrimination, historical trauma, geographic location, and tobacco product sales being a key component of tribal economies [8,9,10,11,12]. There is limited data on the age of smoking initiation among AIs, but the available evidence suggests that AI youth are at particularly high risk for early cigarette smoking compared to other ethnic groups [13,14,15,16,17]. 

Cigarette smoking typically begins during adolescence and the earlier age of initiation portends persistent smoking, higher nicotine addiction, and less success with smoking cessation [18,19,20,21]. In more recent decades AIs, like NHWs, are beginning to smoke at an earlier age [17,22,23,24]. However, age of initiation is not consistent across AI communities, reflecting a complex pattern wherein age of initiation varies by region and gender [17]. For example, AI females living in the Southwest are less likely to initiate smoking by age 18 compared to their male counterparts while, in the Northern Plains, the age of initiation is similar between males and females [17]. 

The primary aim of this study is to determine if age of smoking initiation among groups of AIs has changed over time; and to compare age of smoking initiation in an AI sample with a population-based sample of NHWs. By taking an approach that is historical and within the context of NHW smoking initiation trends, we are able to assemble the most comprehensive portrait of AIs trends in smoking initiation and their changes through the 20th century. This study offers new information as to the smoking initiation trends in a group of AIs and how gender and location may relate to cigarettes use in AI communities. 

## 2. Methods

### 2.1. Sample

The Strong Heart Study (SHS) is the largest epidemiologic investigation of cardiovascular disease in AI communities ever conducted. It was initiated in 1988 to measure risk factors for cardiovascular disease in 4,549 AIs who were members of 13 tribes in Arizona, Oklahoma and North and South Dakota. Referred to as Phase I, the 1988 study was followed by the Strong Heart Family Study (SHFS) or Phase IV in 2001. This phase extended the original cohort study to include 94 families and 3,776 family members. For the present analysis we used data from the first Phase of data collection for each cohort: Phase I for the original cohort and Phase IV for the family study cohort. The dataset included adult participants born as early as 1904 and as recent as 1985, allowing us to have a sample of wide age ranges and a well-defined sample of smoking initiation in AI living in these regions.

The National Health Interview Survey (NHIS) collects self-reported information on health and risk factors by telephone interview based on a random probability sample of the U.S. population. The NHIS uses a complex sampling design that when weighted appropriately produces estimates that can be generalized to the U.S. population. For birth cohort comparability to the SHS, we used person-level data from 30,852 adults interviewed in the 2003 NHIS. We restricted analyses to 19,747 NHW participants in the 2003 NHIS with complete data for smoking history. It was not possible to discern the specific geographic location of the NHIS participants beyond broad census regions. 

### 2.2. Measures: Smoking Status and Age of Initiation

The SHS classified s*moking status* among participants based on asking participants about their experience with tobacco smoking. In Phase I, ever-smoking status was assessed by asking “Have you smoked at least 100 cigarettes in your entire life?” In Phase IV, ever-smoking status was assessed by asking “During your lifetime have you smoked 100 cigarettes or more total?” Response options for both questions included “Yes” or “No.”.

In both Phases I and IV, the *age of smoking initiation* was asked of those who indicated they had smoked at least 100 cigarettes in their lifetime. In Phase I, age of initiation was measured with “How old were you when you first started smoking cigarettes fairly regularly?” In Phase IV, age of initiation was measured with “How old were you when you first started smoking regularly?” Participants responded to both questions with numerical age. 

The NHIS questionnaire asked participants, “Have you smoked at least 100 cigarettes in your entire life?” Participants who responded “Yes” were also asked “How old were you when you first started to smoke fairly regularly?” Birth year was calculated from age and interview year (2003). 

### 2.3. Analysis

All SHS analyses were stratified according to region: Arizona, Oklahoma, and the Dakotas. A descriptive analysis was performed for all variables, examining mean and standard deviation for continuous variables and percent for categorical variables. We plotted the cumulative incidence of smoking initiation by age 18 years according to age of initiation overall and stratified by sex. The log-rank test was used to compare the cumulative smoking initiation curves according to age in the three SHS regions and across sex within each region. 

We assessed the association between age of smoking initiation and birth cohort by examining the proportion of participants who initiated smoking by age 18 for eight birth cohorts. Age 18 is used as the cutoff here because it has been shown as a significant discriminant for early smoking [25,26]. Smoking onset certainly occurs after age 18, but it is understood as leading to a lower nicotine dependency and is treated differently in the literature [19,24,27]. The birth cohorts were divided by decade to include pre-1920, 1920–1929, 1930–1939, 1940–1949, 1950–1959, 1960–1969, 1970–1979, and 1980–1985. We used binary logistic regression to conduct a test for a monotonic increasing trend in proportion of participants who initiated smoking by age 18 according to birth cohort. The outcome variable was an indicator of smoking initiation by age 18 years and the independent variable was ordered birth cohort. Finally, we used Cox proportional hazards regression modeling to examine the influence of birth cohort on rate of smoking initiation by age 18. Birth cohort was included in the model using dummy variables, with the oldest birth cohort, pre-1920s, serving as the reference. We also fit a Cox regression model that included a single ordered birth cohort variable to test for a monotonic trend in the hazard ratio for smoking initiation by age 18 according to birth cohort. Results are presented as hazard ratios with 95% confidence intervals that show the precision of the estimate for each birth cohort. The test for trend examines if there is a monotonic increase or decrease in the hazard ratios across all cohorts. Models that included an interaction term for birth cohort and sex also were fit to formally test if the age of smoking initiation differed in males and females across birth cohorts. 

Using the 2003 NHIS data on age at smoking initiation we generated comparable birth cohort and sex-specific analyses in the NHW population. The NHIS analyses used the appropriate sampling weights to obtain population estimates. However, because of the differences in sampling design we did not formally perform statistical tests directly comparing SHS and NHIS results. 

All analyses were performed using IBM SPSS Statistics version 20 and a significance level of 0.05 was considered the threshold for statistical significance. 

## 3. Results

SHS participants included more females than males and nearly half of all participants were born between 1930 and 1949 (Table 1). Education varied between regions with Oklahoma having the greater level of high school graduates (75.1%) than other areas. The percent who initiated smoking by age 18 differed across the regions with the highest percent found in the Dakotas (50.1%), followed by Arizona (40.8%) and Oklahoma (38.2%). When compared to AI from the SHS, a larger portion of NHWs belonged to more recent birth cohorts. High School graduation rates were higher among NHW than AI and a smaller proportion started smoking regularly by age 18 (32.9%).

**Table 1:** Participant characteristics from the Strong Heart Study (SHS) and the 2003 National Health Interview Survey (NHIS).

	*American Indians*			*Non-Hispanic whites*
Variable	SHS-AZ	SHS-OK	SHS-N/SD	NHIS
	(n = 2,343)	(n = 2,327)	(n = 2,403)	(n = 19,747) *
Age, *mean years (SE)*	48 (0.3)	51 (0.3)	49 (0.3)	47 (0.2)
Birth cohort, *%*				
1910s and before	3	5	4	3
1920–1929	14	17	15	8
1930–1939	24	24	26	10
1940–1949	23	23	22	15
1950–1959	9	10	8	20
1960–1969	11	11	11	19
1970–1979	11	8	10	16
1980–1985	6	3	5	9
Female, *%*	63	58	58	52
High school graduate, *%*	41	75	60	88
Smoker by age 18 years, *%*	41	38	50	33

AZ = Arizona, OK = Oklahoma, N/SD = North/South Dakota, SE = standard error. *: Unweighted sample size.

**Figure 1:**
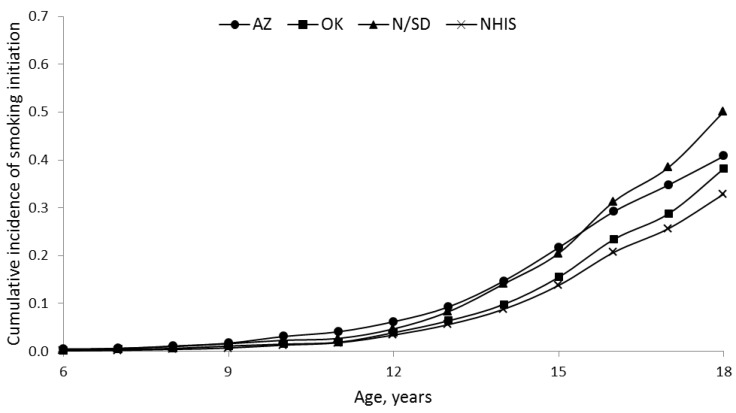
Cumulative incidence of smoking initiation across ages 6 to 18 years in American Indians from the Strong Heart Study (SHS) and non-Hispanic whites from the National Health Interview Survey (NHIS). AZ = Arizona, OK = Oklahoma, N/SD = North/South Dakota.

**Figure 2:**
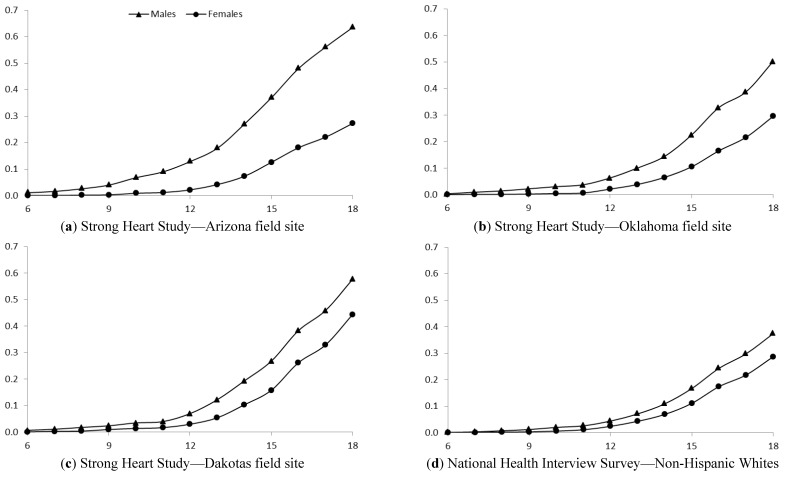
Cumulative incidence of smoking initiation across ages 6 to 18 years in American Indians from the Strong Heart Study and non-Hispanic whites from the National Health Interview Survey, stratified by sex.

Figure 1 shows the cumulative incidence of smoking initiation by age 18 was highest in the Dakotas and lowest in NHWs. A cumulative incidence shows the proportion of a population who experience an event (initiate smoking) over a specified period of time (ages 6 to 18). The incidence curves for age of initiation were significantly different in the three SHS regions (*p* < 0.001). The cumulative incidence of smoking initiation by age 18 was higher in males than females in all SHS regions and in NHWs (Figure 2, all *p* < 0.001). Table 2 shows the association between smoking initiation by age 18 and birth cohort according to sex. Arizona and Dakota male SHS participants demonstrated no consistent trend in the proportion initiating smoking by 18 according to birth cohort (*p* = 0.17 and 0.68, respectively). Among SHS participants in Oklahoma and among NHW, males appear to be initiating smoking by age 18 less frequently in more recent birth cohorts (*p* = 0.05 and <0.001, respectively). Conversely, in both the SHS sample and NHW sample the percent of females initiating smoking by 18 has increased in more recent birth cohorts (all *p* < 0.001). Female SHS participants from the Dakota regions had the largest absolute change, rising from 28.1% in those born before 1920 to 72.1% in the 1980–1985 birth cohort. NHW females had the lowest absolute change, increasing from 12.0% in the earliest birth cohort to 31.0% in the most recent cohort. 

**Table 2:** Percent of Strong Heart Study (SHS) and National Health Interview Survey (NHIS) participants with smoking initiation age 18 years or younger according to sex.

	*American Indians*			*Non-Hispanic whites*
Birth cohort	SHS-AZ%	SHS-OK%	SHS-N/SD%	NHIS%
	(95% CI)	(95% CI)	(95% CI)	(95% CI)
*Males*				
Before 1920	50 (24–76)	52 (37–68)	50 (31–69)	32 (26–39)
1920–1929	61 (52–70)	57 (49–65)	50 (43–58)	48 (44–53)
1930–1939	65 (59–72)	44 (38–51)	62 (56–67)	45 (42–49)
1940–1949	63 (57–69)	51 (45–58)	63 (57–69)	45 (42–48)
1950–1959	67 (57–77)	52 (41–63)	51 (39–64)	38 (36–41)
1960–1969	63 (53–72)	34 (25–44)	46 (36–56)	31 (29–34)
1970–1979	56 (46–66)	47 (36–58)	50 (40–59)	34 (31–37)
1980–1985	49 (35–63)	48 (31–64)	67 (54–79)	29 (25–33)
*p*_trend_	0.17	0.05	0.68	<0.001
*Females*				
Before 1920	12 (2–22)	14 (5–23)	28 (16–40)	12 (9–16)
1920–1929	13 (9–17)	20 (15–25)	33 (27–40)	19 (16–21)
1930–1939	20 (16–24)	20 (15–24)	37 (32–42)	23 (21–26)
1940–1949	29 (24–34)	36 (30–41)	42 (37–48)	27 (25–30)
1950–1959	35 (26–43)	30 (23–38)	51 (42–60)	31 (29–33)
1960–1969	40 (32–47)	41 (33–49)	53 (4–60)	33 (31–36)
1970–1979	39 (31–47)	38 (29–48)	55 (46–63)	33 (31–36)
1980–1985	33 (23–43)	43 (26–59)	72 (61–84)	31 (27–35)
*p*_trend_	<0.001	<0.001	<0.001	<0.001

AZ = Arizona, OK = Oklahoma, N/SD = North/South Dakota.

Hazard ratios for the association of birth cohort with the age of smoking initiation by sex and region are shown in (Table 3). Male SHS participants from the Dakotas appear to initiate smoking at similar ages across the birth cohorts. This differs from SHS male and female participants in Arizona and Oklahoma, and NHW males in more recent birth cohorts, who were initiating smoking at a later age compared to those born in earlier birth cohorts. For example, SHS participants from the Oklahoma region in the 1980–85 birth cohort were 0.7 times as likely to initiate at a younger age compared to those born prior to 1920 (HR = 0.7, 95% CI = 0.4–1.3). This association was marginally significant for SHS participants from Arizona (*p* = 0.06) and reached statistical significance for SHS participants of the Oklahoma region and NHW males (*p* = 0.02 and <0.001, respectively). Conversely, both SHS participants and NHW females were initiating smoking at an earlier age in more recent birth cohorts compared to those born in older birth cohorts (all *p* < 0.001). For example, SHS female participants from the Dakotas in the 1980–85 birth cohort were almost four times as likely to initiate smoking at a younger age compared to those born prior to 1920 (HR = 3.9, 95% CI = 2.2–6.9). Similarly, NHW females in the 1980–1985 birth cohort were three times as likely to initiate smoking at a younger age compared to those born prior to 1920 (HR = 3.0, 95% CI = 2.1–4.2). 

**Table 3:** Hazard ratios (HRs) for smoking initiation by age 18 years or younger according to sex in Strong Heart Study (SHS) and National Health Interview Survey (NHIS) participants.

	*American Indians*			*Non-Hispanic whites*
Birth cohort	SHS-AZ	SHS-OK	SHS-N/SD	NHIS
	HR (95% CI)	HR (95% CI)	HR (95% CI)	HR (95% CI)
*Males*				
Before 1920	1.0	1.0	1.0	1.0
1920–1929	1.1 (0.6–2.2)	1.0 (0.6–1.6)	1.0 (0.6–1.8)	1.6 (1.2–2.1)
1930–1939	1.3 (0.7–2.6)	0.7 (0.5–1.2)	1.3 (0.8–2.3)	1.5 (1.1–2.1)
1940–1949	1.2 (0.6–2.4)	0.9 (0.6–1.4)	1.4 (0.8–2.4)	1.5 (1.1–2.0)
1950–1959	1.4 (0.7–2.8)	0.9 (0.5–1.5)	1.1 (0.6–2.0)	1.2 (0.9–1.6)
1960–1969	1.1 (0.6–2.2)	0.5 (0.3–0.9)	0.9 (0.5–1.6)	0.9 (0.7–1.3)
1970–1979	0.9 (0.5–1.9)	0.8 (0.4–1.3)	1.1 (0.6–1.9)	1.1 (0.8–1.4)
1980–1985	0.8 (0.4–1.7)	0.7 (0.4–1.3)	1.6 (0.9–2.9)	0.9 (0.6–1.2)
*p*_trend_	0.06	0.02	0.96	<0.001
*Females*				
Before 1920	1.0	1.0	1.0	1.0
1920–1929	1.1 (0.4–3.0)	1.5 (0.7–3.0)	1.3 (0.7–2.2)	1.6 (1.1–2.3)
1930–1939	1.8 (0.7–4.4)	1.5 (0.7–2.9)	1.4 (0.8–2.4)	2.0 (1.4–2.9)
1940–1949	2.9 (1.2–7.1)	3.0 (1.5–6.0)	1.7 (1.0–2.8)	2.5 (1.8–3.5)
1950–1959	3.6 (1.4–9.0)	2.5 (1.2–5.0)	2.1 (1.2–3.6)	2.9 (2.1–4.0)
1960–1969	4.0 (1.6–10.0)	3.5 (1.7–7.0)	2.3 (1.3–3.9 )	3.2 (2.3–4.5)
1970–1979	3.9 (1.6–9.8)	3.2 (1.6–6.6)	2.6 (1.5–4.4)	3.3 (2.4–4.5)
1980–1985	3.3 (1.3–8.5)	3.6 (1.6–8.0)	3.9 (2.2–6.9)	3.0 (2.1–4.2)
*p*_trend_	<0.001	<0.001	<0.001	<0.001

AZ = Arizona, OK = Oklahoma, SD = South Dakota.

## 4. Discussion

We found that the incidence of smoking initiation by age 18 years among a well-defined and geographically diverse sample of AIs varies by sex, birth cohort and geographic region. Overall, the proportion of AI females from this sample who initiate smoking by age 18 has significantly increased, while there has been little change or even a slight decrease in the proportion for males. By the end of the 20th century, the incidence of smoking initiation by age 18 appears to be similar for both female and male AI from this sample. 

**Figure 3:**
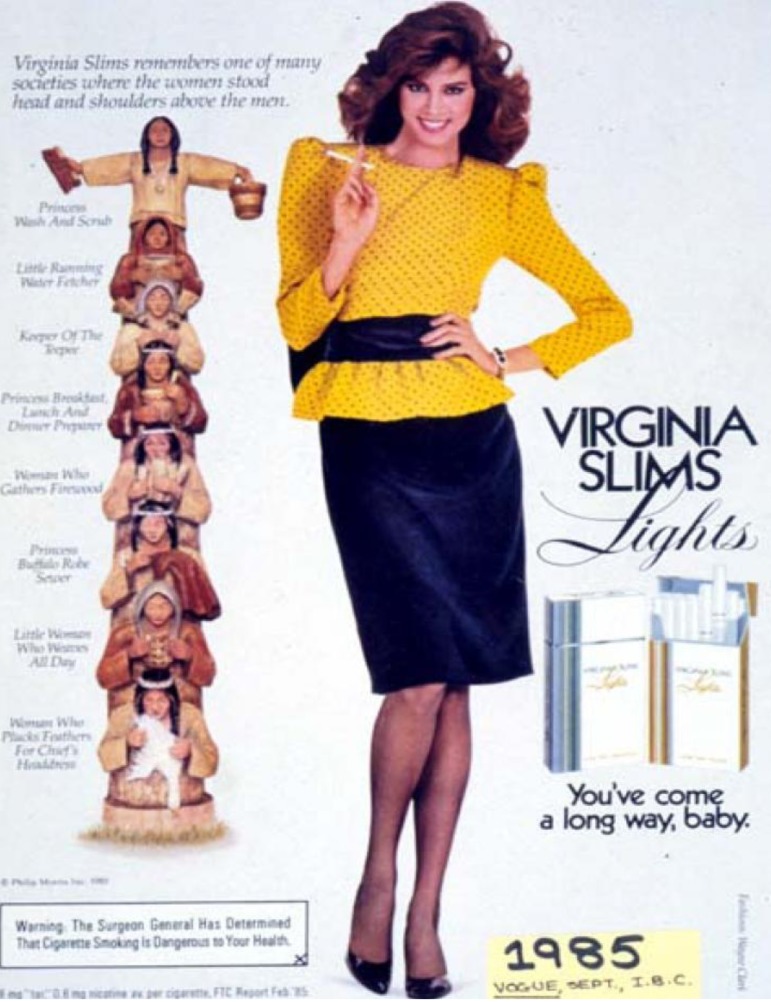
Virginia Slims’ advertisement in *Vogue* magazine in 1985 depicting a professionally dressed woman smoking next to traditionally (and stereotypically) dressed American Indian women stacked as a totem pole while engaged in various tasks.

This apparent decrease in age of smoking initiation for all AI female SHS participants is a cause for concern. Early age of smoking initiation is a risk factor for persistent smoking beyond adolescence, and smoking cessation is more challenging among those who start smoking earlier. Our study indicates that women participants from the SHS mirror the national shift in women’s smoking habits over time to a younger age of initiation. The causes are complex, but are likely in part the result of greater economic independence of women [28,29] and societal change in substance use by women [30]. In particular, an alteration in gender norms led to the increase in female smoking over the later 20th century [30]. The women’s liberation movement in the 1960s and 70s, along with greater economic independence, were subverted by the tobacco companies to present an idealized version of the modern woman as a smoker [31,32]. The iconic Virginia Slims tobacco advertisements, symbolized by the theme, “You’ve come a long way, baby,” presented images of an independent, healthy and upwardly mobile woman who naturally would smoke a stylish cigarette [33]. Figure 3 is an example this marketing campaign aimed toward women in this period where Virginia Slims incorporates AI themes into women’s empowerment and declares that the brand “remembers one of many societies where the women stood head and shoulders above the men.” These ads sought to capitalize on women’s increasing empowerment. Our findings suggest that AI females of that generation were not immune to social shifts during this period.

Another possible explanation for the increase in women’s smoking over the 20th century is the development of filtered cigarettes. It was not until the 1950s when filters were added to widely marketed cigarettes; by the 1960s, filtered cigarettes dominated the market with the perception that they provided a “healthier” alternative to unfiltered varieties [34]. Women adopted filtered cigarettes before men [30], which may indicate that women’s taste and self-image, even pre-women’s empowerment, were less compatible with older forms of tobacco consumption such as pipes, cigars and smokeless tobacco [35]. The addition of filters also may explain the sharply gendered division around these older forms of tobacco that still persists today. 

We confirm the previous findings by Nez Henderson *et al*. that AIs in the Dakotas have high rates of smoking initiation by age 18. Previous studies on AI smoking in the Dakotas using a slightly different definition of smoking initiation showed no difference in smoking initiation across gender [17]; however, we observed that males were more likely to initiate by age 18 compared to females. Our Dakotas findings were also very similar to the NHW results among the earliest birth cohorts. This might reflect the concentrated ceremonial use of tobacco among tribes in the Northern Plains that dates back centuries [36,37]. Though ceremonial uses of tobacco vary by tribe and practice, tobacco has had a longstanding presence in this region. The high traditional use of tobacco in the region was relatively constant through the 20th century and offers insight into the persistently high rates in the Dakotas over this period. Paradoxically, the cultural remoteness that insulated Arizonan females from early smoking, may have sustained the more prevalent traditional tobacco use among the Dakota that contributed to the increase in early smoking and helped it persist. 

Initiating tobacco use by 18 years of age gradually declined among male SHS participants from Oklahoma across the decades. Why this occurred is not entirely clear. One possible reason is income. Oklahoma AI appear to have higher levels of socio-economic status group when compared to AI populations in other regions [38]. Higher socioeconomic status is negatively associated with adult smoking [39,40,41,42] and would reduce the smoking rates of children of the better-off [43]. Greater frequency of high school completion among Oklahoma SHS participants throughout the 20th century may have contributed to the decline in early male initiation through the influence of parental smoking behavior. Since higher educational attainment reduces the risk of smoking by adults, [44,45,46] and parental smoking status in turn influences children’s rates [47,48,49], it is possible that the greater frequency of completing high school among Oklahoma SHS participants reduced parental smoking. This subsequently could have led to a lower likelihood of smoking among their children. In Native communities, this pattern is replicated among those with greater education, such as Oklahoma AIs, smoking at lesser rates than poorer members of the group [50]. Though not assessed in the SHS study, parental and family smoking status among participants deserves closer examination as it is understood as being a determinant of smoking risks [49]. Another explanation may lie in the observation that the Oklahoma population had the highest high school completion rate compared to the other SHS centers. At first glance, the percentage of high school graduates of each population might seem unrelated to early smoking, as most people younger than 18 years of age would not yet be high school graduates. However, multiple studies confirm that dropping out of high school increases the risk of smoking as well as other forms of substance abuse [44,45,46]. Participating in after-school programs and achieving high grade point averages—two characteristics only possible when enrolled in high school–protect against early smoking [44,46]. Time spent completing a high school diploma could have allowed more Oklahoma SHS male participants to participate in extracurricular activities such as athletic programs that reduce the likelihood those adolescents start smoking. 

Attitudes toward tobacco use also may have contributed to the changes in early initiation rates for SHS participants from the Oklahoma region. Comparing from a sample of AI residing in Oklahoma revealed attitudes comparable with those of the general US population, one study found an overlap in beliefs around the health consequences of using tobacco between the two groups. Attitudes toward tobacco’s addictiveness and dangers are known to influence smoking behaviors [51,52]. Similar attitudes between Oklahoma SHS participants and the general population about smoking would be an additional explanation for why age of initiation among the former is closer to that of NHWs than the SHS participants from other regions. 

The local ecology of tobacco sales and regulation on tribal land also may play a role in the changing patterns of tobacco use initiation. In 1976, the United States Supreme Court confirmed that Indian tribes enjoy a unique tax status that exempts cigarette sales to tribal members on reservation lands from state taxation. Cigarettes sold to non-members are subject to state taxation [53]. The Supreme Court, however, constrained state officials from entering tribal member-owned or tribal government-owned cigarette retail facilities to seize cigarettes sold absent remittance of state taxes [54]. Consequently, non-tribal members purchase tribally sold cigarettes at a cheaper price, tribal revenues increase and the retail outlets, known as “smoke shops,” provide much needed jobs on the reservation. The presence of low-cost cigarettes is particularly hazardous to younger smokers as “pack price” is one of the greatest determinants of use [55]. The less regulated tobacco environment allows the tobacco industry to have a high presence at AI rodeos and pow-wows whereby AIs were continually exposed to cigarette advertisements. The unanticipated consequences are that reservations are awash in inexpensive cigarettes and saturated by advertisements promoting positive images of tobacco use, the combination of which encourages early initiation of smoking [56].

There are limitations to our data and measures of smoking initiation among the SHS participants. We restricted our analysis to smoking by age 18, which misses later onset smoking. The description of AI smoking trends is therefore limited and based only on the sample attained from the SHS. It is noted here that there are over 550 distinct AI tribes in the United States with many cultural, linguistic and geographical differences, though despite these distinctions earlier smoking is understood as being a common source of the more pernicious forms of cigarette dependence for AIs [19,24,27]. The precise definition of what constitutes a “regular smoker” is an unresolved question in the literature [57,58]. A different criterion may have produced different results. We used retrospective recall for age of smoking initiation that, for most of our participants, occurred decades ago. Previous work with other population samples has demonstrated that this method is accurate, but there are no studies of retrospective recall for age of smoking initiation among AIs [45,59]. A similar measurement challenge applies to determining when an individual “becomes” a smoker [60,61]. Some studies equate age at initiation with the first cigarette, whereas others use the first sustained period of smoking. Tobacco is smoked and used in non-smoking ways by AI communities in rituals and ceremonies, which may further complicate this issue. However the SHS attempted to carefully distinguish between commercial and ceremonial use in identifying a regular smoker. 

Other factors than those available through the SHS are potentially related to the initiation of smoking. Examples include family history of smoking, extent of smoking among one’s peers, personality attributes (e.g., sensation-seeking), and adolescent psychological factors (e.g., anxiety, hostility) [46,48,62,63]. Finally, we were most interested in the overall trend for age of smoking initiation across all birth cohorts; however, focus on the overall trend may miss deviations that occur in the intermediate birth cohorts. Despite these limitations, the size of the SHS sample, and its geographic scope, do offer a good preliminary opportunity to describe changes in smoking behavior in a distinct group of AIs in comparison with NHWs.

## 5. Conclusions

The historical perspective that this study offers may enable us to better understand the social and policy changes that could impact smoking initiation trends. Smoking continues to be a major public health problem and foreshadows serious tobacco-related morbidities and mortality. Recent work suggests a number of promising interventions for altering smoking patterns and stopping or delaying the onset of smoking [64,65,66]; application of these techniques needs to be considered in accordance with the age, gender, regional, and cultural variation evident in these findings. If adolescent smoking is not reduced, all populations including AI will further be burdened by the costs associated with chronic tobacco related impairments. This health crisis created by tobacco use has the potential to offset the social, political and economic gains many of AI communities have recently experienced [67]. We strongly urge further research in this area especially that which promises greater insight into the historical, economic, and policy forces at play.
